# Novel albumin-binding multifunctional probe for synergistic enhancement of FL/MR dual-modal imaging and photothermal therapy

**DOI:** 10.3389/fchem.2023.1253379

**Published:** 2023-08-01

**Authors:** Cheng Yu, Zhuyuan Ding, Huan Liu, Yulu Ren, Minping Zhang, Qiuling Liao, Tao Luo, Lujing Gao, Shiyi Lyu, Huiwen Tan, Linan Hu, Zhu Chen, Pengfei Xu, Enhua Xiao

**Affiliations:** ^1^ Department of Radiology, The Second Xiangya Hospital, Central South University, Changsha, Hunan, China; ^2^ Department of Radiology, Zhuzhou Central Hospital, Zhuzhou, Hunan, China; ^3^ Translational Pharmaceutical Laboratory, Jining First People’s Hospital, Shandong First Medical University, Jining, China; ^4^ Institute of Translational Pharmacy, Jining Medical Research Academy, Jining, China

**Keywords:** drug delivery, albumin, fluorescence imaging, MRI, photothermal therapy

## Abstract

The fluorescence/magnetic resonance (FL/MR) dual-modal imaging could provide accurate tumor visualization to guide photothermal therapy (PTT) of cancer, which has attracted widespread attention from scientists. However, facile and effective strategies to synergistically enhance fluorescence intensity, MR contrast and photothermal efficacy have rarely been reported. This study presents a novel multifunctional probe Gd-EB-ICG (GI) for FL/MR dual-modal imaging-guided PTT of cancer. GIs can self-assemble with endogenous albumin to form drug-albumin complexes (GIAs), which exhibit excellent biocompatibility. Albumin can protect GIAs from the recognition and clearance by the mononuclear phagocytic system (MPS). High plasma concentration and long half-life allow GIAs to accumulate continuously in the tumor area through EPR effect and specific uptake of tumor. Because of the prolonged rotational correlation time (τR) of Gd chelates, GIAs exhibited superior MR contrast performance over GIs with more than 3 times enhancement of longitudinal relaxation efficiency (r_1_). The fluorescence quantum yield and photothermal conversion efficiency of GIAs was also significantly improved due to the constrained geometry, disrupted aggregation and enhanced photothermal stability. This simple and feasible strategy successfully resulted in a synergistic effect for FL/MR dual-modal imaging and photothermal therapy, which can cast a new light for the clinical translation of multifunctional probes.

## Introduction

Cancer is a leading cause of death and accounted for almost 10.0 million deaths worldwide in 2020 (Bray et al., 2021; Sung et al., 2021). Developing precise and efficient theranostic techniques is particularly relevant to improve the longevity of cancer patients. Currently, a variety of nano carriers equipped with imaging and treatment components have been developed for precise diagnosis and imaging guided therapy (Wang M. et al., 2020; Wang Y. et al., 2020). However, the complex processes, host foreign body responses, and potential long-term toxicity seem to limit their clinical application (Larsen et al., 2016a). Endogenous albumin is an attractive next-generation drug delivery carrier.

Human serum albumin (HSA) is the most abundant plasma protein with a half-life of approximately 19 days (Anderson et al., 2006; Kratz, 2008). HSA exhibits a molecular weight of 66.5 kDa and an effective diameter of about 7.2 nm (Garcovich et al., 2009). HSA contains multiple hydrophobic binding pockets and naturally serves as a transporter of a number of different ligands. The negative charge on the surface of HSA makes it highly water-soluble (Larsen et al., 2016a). Notably, albumin specifically targets tumor regions due to its enhanced permeability and retention (EPR) effect, abnormal nutritional needs, albumin receptor binding, and SPARC-inducing effect (Neuzillet et al., 2013; Larsen et al., 2016b; Liu and Chen, 2016). These properties as well as its ready availability, biodegradability, and lack of toxicity and immunogenicity make it an ideal candidate for drug delivery. Additionally, albumin has unexpected effects as a drug delivery carrier.

In diagnostic imaging, a single imaging technique often cannot provide effective and accurate information for clinical diagnoses and medical research (Louie, 2010). The integration of MRI and FLI can overcome the limitations of each other and achieve complementary advantages, offering more detailed anatomical or biological tumor information (Rosa et al., 2015; Yan et al., 2017). Furthermore, conventional clinical treatments for cancer, including surgical intervention, chemotherapy, and radiotherapy, have major drawbacks (Cihoric et al., 2015). As a novel non-invasive cancer treatment strategy, photothermal therapy (PTT) has attracted extensive attention owing to its high efficiency, easy operation, negligible side effects, and good bioavailability (Li et al., 2017; Wu et al., 2017). Importantly, PTT can ignore cellular resistance as it induces cell death via physical mechanisms, such as protein denaturation and membrane rupture (Valcourt et al., 2019). Nevertheless, simple and effective strategies to synergistically enhance fluorescence intensity, MR contrast, and photothermal conversion efficiency have rarely been reported.

As a multifunctional probe delivery carrier, HSA can be used for the synergistic enhancement of FL/MR dual-modal imaging and photothermal therapy. Small molecule Gd chelates are the most commonly used contrast agents in clinical practice. Generally, connecting the ligand with macromolecules is an effective strategy to enhance the performance of Gd-based contrast agents (Werner et al., 2007; Song et al., 2008; Yang et al., 2008; Mastarone et al., 2011). As a natural macromolecular substance in the human body, HSA can effective limit the rotation of gadolinium chelates in the magnetic field, thereby prolonging the rotational correlation time (τR), resulting in a sharp increase in the longitudinal relaxivity (r_1_) (Chen et al., 2011; Poeselt et al., 2012). Indocyanine green (ICG) is the only near-infrared (NIR) dye approved for clinical application by the FDA. ICG has good optical properties and thermotherapy ability, which can be applied for NIR imaging and photothermal therapy (PTT) (Haller et al., 1992). Recent studies have demonstrated that ICG emits tail fluorescence in the NIR-II window, which can be used for NIR-II imaging (Bhavane et al., 2018; Carr et al., 2018). The intercalation of ICG into the HSA pocket may lead to emission enhancement due to constrained geometry and disruption of aggregation (Tian et al., 2019; Yue et al., 2022). In addition, HSA can also enhance the photothermal conversion efficiency of ICG by improving its photothermal stability (Yu et al., 2021).

**SCHEME 1:**
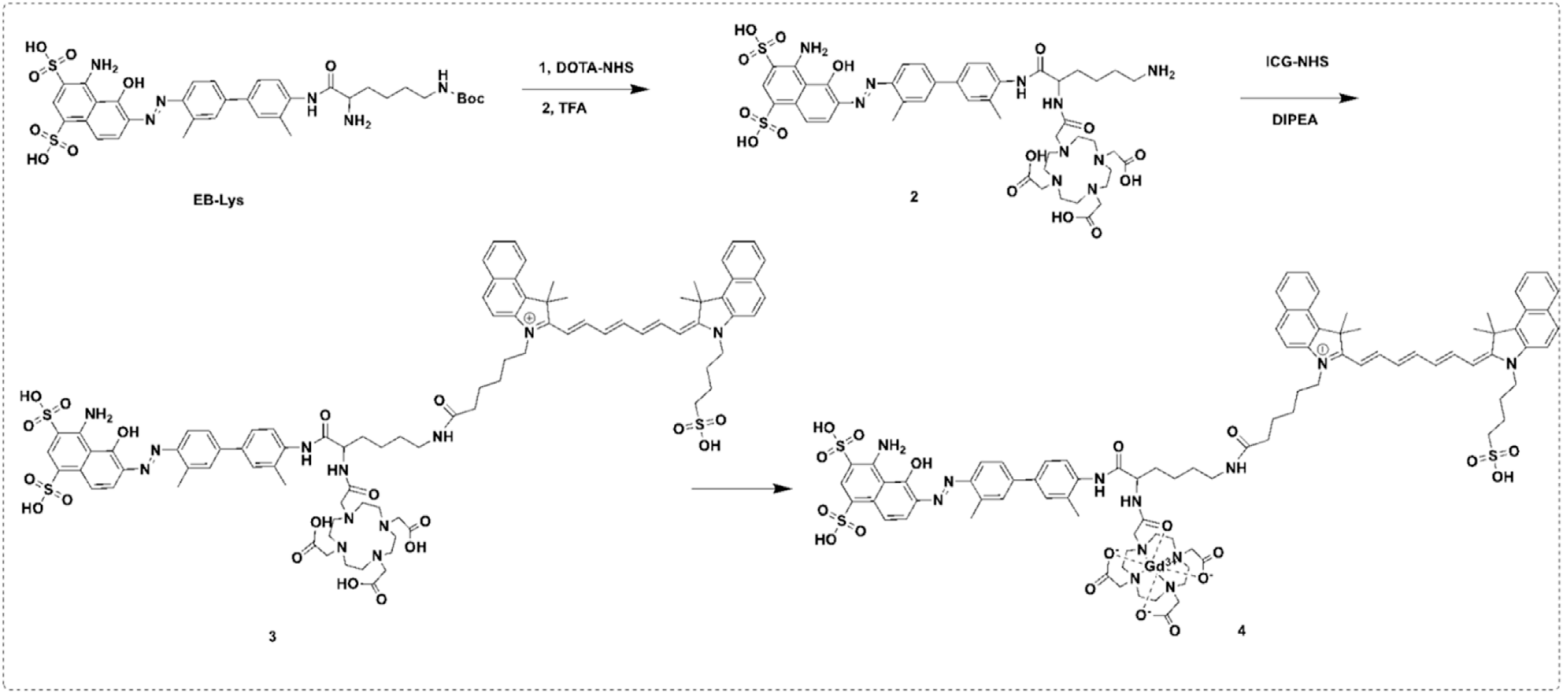
The synthesis of the GI.

**SCHEME 2:**
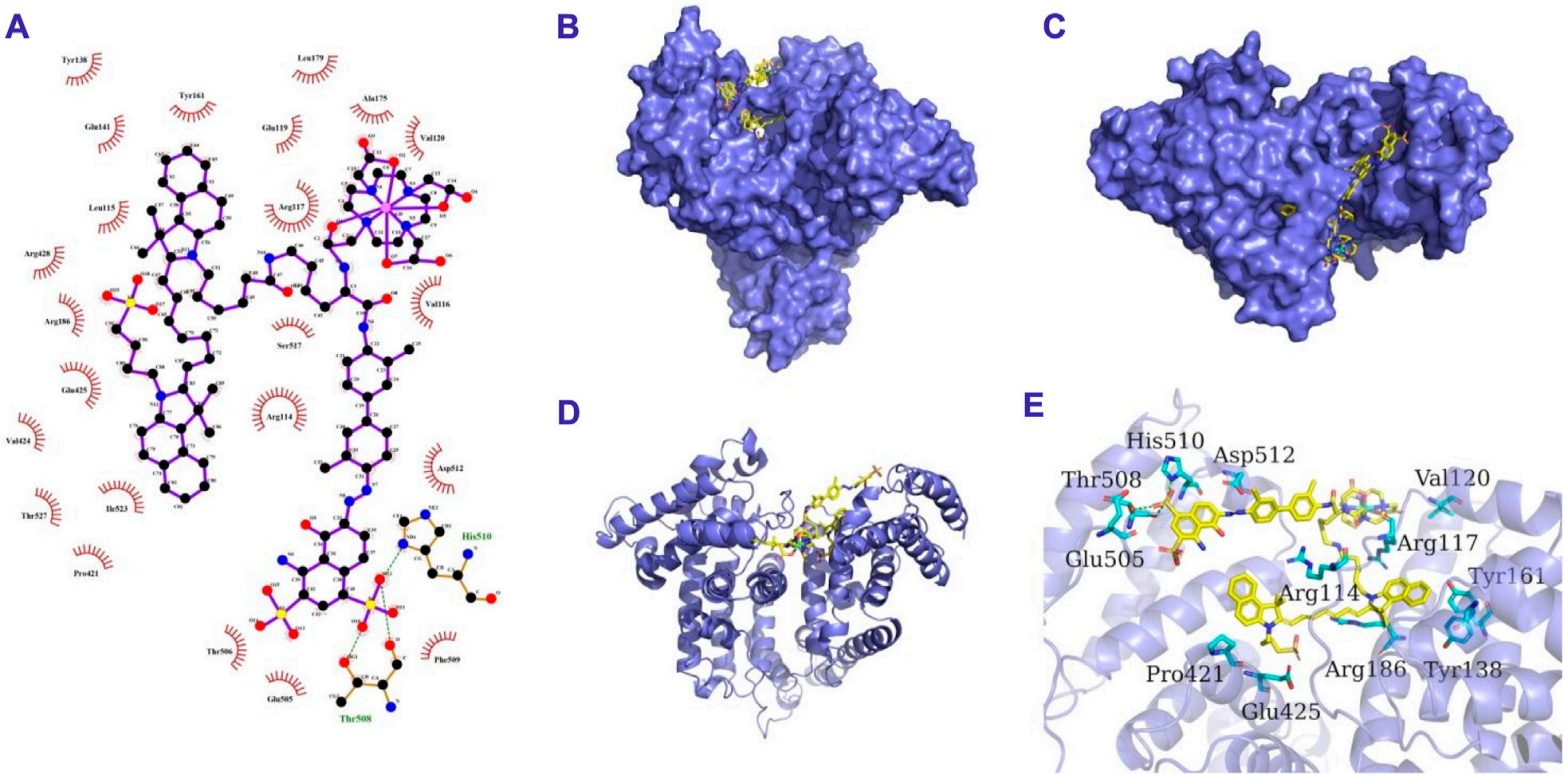
The 2D **(A)**, surface **(B,C)**, cartoon **(D)** and 3D **(E)** binding model of GI and HSA. GI is colored in yellow. The cartoon and surface of HSA is colored in slate. The residues in HSA are shown as cyan sticks. The hydrogen bond interactions are depicted as green lines.

Evans Blue (EB) is an azo dye with high serum albumin binding affinity (Xie et al., 2016). There are approximately 14 binding sites on albumin for EB (Niu et al., 2014; Liu and Chen, 2016). This study outlines a simple and feasible method to construct a novel multifunctional probe, Gd-EB-ICG (GI), was designed and synthesized by a facile and feasible method (Scheme 1). The probe is comprised of three parts: 1) Gd-DOTA for MR imaging; 2) ICG for NIR-I/NIR-II imaging and photothermal therapy; 3) EB for albumin binding. When injected intravenously into the blood circulation, GIs rapidly assemble with albumin into drug-albumin complexes (GIAs), whose molecular docking model is shown in Scheme 2. GIAs can evade the recognition and clearance of the mononuclear phagocytic system (MPS) and thus obtain a very long circulation time. High plasma concentration and long half-life allow GIAs to accumulate continuously in the tumor area through EPR effect and specific uptake of tumor. *In vitro* experiments demonstrated that GIAs exhibited superior fluorescence quantum yield, r_1_ and photothermal conversion efficiency than GIs. *In vivo* experiments demonstrated that GIAs exhibits excellent tumor aggregation, imaging and killing effects. This facile and feasible strategy not only achieved effective drug delivery, but also successfully realized a synergistic effect for FL/MR dual-modal imaging and photothermal therapy, which can cast a new light for the clinical translation of multifunctional nanoprobes.

## Results and discussion

### Synergetic enhancement of optical/magnetic and thermal properties of GIAs in solution

**FIGURE 1:**
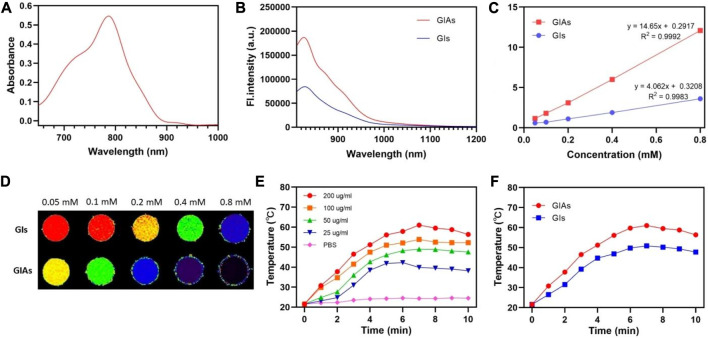
**(A)** UV-Vis absorption spectra of GIAs. **(B)** Emission spectra of GIAs and GIs. **(C)** T1 relaxation properties and **(D)** corresponding phantom images of GIAs and GIs. The color from blue to red was used to indicate the increase in T1 relaxation time. **(E)** Temperature of GIAs at different concentrations following laser irradiation for 10 min. **(F)** Increasing photothermal temperature curves of GIAs and GIs with irradiation time.

The optical properties of GIAs were investigated by UV-visible absorption and fluorescence spectroscopy. As shown in Figures 1A, B, the UV-vis absorption spectrum of GIAs showed a maximum peak at 800 nm, while the emission spectrum exhibited a maximum peak at 830 nm. GIAs exhibited superior fluorescence quantum yield estimated by the area under the curve over GIs with more than 2 times enhancement. The enhanced emission can be explained by two events: constrained geometry and disruption of aggregation, which reduces non-radiative decay. In addition, GIAs emits tail fluorescence in the NIR-II window, demonstrating its usefulness for NIR-II imaging.

The longitudinal (T_1_) relaxation times were measured using a 3.0 T MRI scanner to evaluate the MR imaging capacity of GIAs as an effective T_1_-weighted MRI contrast agent. As shown in Figure 1C, GIAs exhibited superior r_1_ (14.65 mM^−1^s^−1^) over GIs (4.06 mM^−1^s^−1^) with nearly four times enhancement. The enhanced r_1_ may be attributed to the bulky and rigid macromolecular structure of albumin. This structure could dramatically prolong the rotational correlation time (τR), resulting in an increase in longitudinal relaxivity, yielding a better MR contrast performance. Furthermore, the phantom images also demonstrated that GIAs exhibited superior MR signal contrast than GIs at the same Gd concentration (Figure 1D).

Subsequently, the photothermal conversion efficiency of GIAs was evaluated by measuring the temperature elevation of GIAs solution after being exposed to an 808 nm laser (0.5 W/cm^2^, 10 min). As shown in Figure 1E, the concentration and irradiation time-dependent temperature of the GIAs solution increased significantly under laser irradiation. Specifically, the GIAs solution (200 μg/mL) showed remarkable and rapid temperature elevation upon irradiation, reaching a maximum temperature rise of 39.4°C (from 21.6°C to 61°C). In contrast, the GIs solution showed limited photothermal conversion efficiency under the same condition (temperature rise 29.3°C) (Figure 1F). The enhanced photothermal conversion efficiency may be attributed to the improved structural rigidity and photothermal stability. All these results indicate that GIAs would has great potential as a high-performance multifunctional probe for synergistic enhanced FL/MR imaging and photothermal therapy.

### *In vitro* cytotoxicity and cellular uptake of GIAs

**FIGURE 2:**
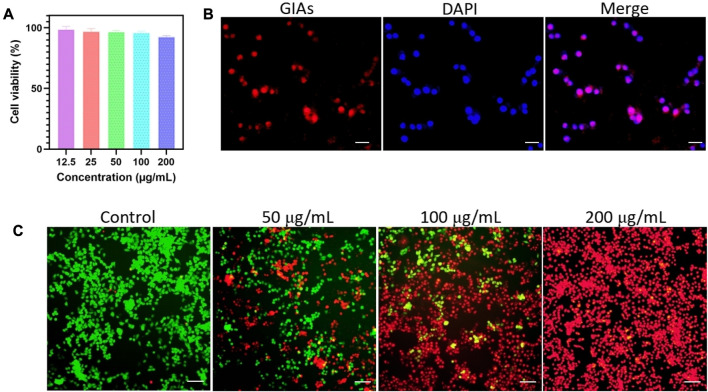
**(A)** Cell viability of 4T1 cells after incubation with GIAs at different concentrations for 24 h. **(B)** Fluorescence microscopy images of 4T1 cells treated with GIAs. Scale bar: 100 μm. **(C)** Fluorescence images of 4T1 cells upon NIR irradiation with different concentrations of GIAs. Green: FDA, live cells; Red: PI, dead cells. Scale bar: 250 μm.

The cytotoxicity experiment was performed on 4T1 cells by CCK-8 assay to evaluate the cytocompatibility of GIAs. As shown in Figure 2A, upon incubation with GIAs for 24 h, the cell viability of 4T1 cells remained >90% at the highest concentration of 200 μg/mL. The results indicated the excellent biocompatibility and very low biotoxicity of GIAs *in vitro*. Moreover, the cellular uptake of GIAs on 4T1 cells was investigated by fluorescence microscopy. The fluorescence microscopy images displayed strong red fluorescence signals in the cytoplasm of 4T1 cells after incubation with GIAs, which perfectly integrated with the blue fluorescence signals of DAPI (Figure 2B). This may be attributed to the albumin receptor (gp60) distributed on cancer cell surfaces, which can bind albumins and complete the transcytosis process (Wang et al., 2021). These results demonstrate that GIAs could effectively and specifically target tumor cells for FL/MR imaging and photothermal therapy.

### *In vitro* PTT of GIAs

The photothermal therapeutic efficiency of GIAs was evaluated by incubating different concentrations of GIAs with 4T1 cells. Fluorescence staining was carried out to observe live cells (FDA, green) and dead cells (PI, red). As shown in Figure 2C, only the green fluorescence signal was observed in the control group, indicating that the cell viability was not compromised after being subjected to irradiation. In contrast, 4T1 cells treated with GIAs showed evident red fluorescence. With increasing concentrations of GIAs, a greater number of dead cells were observed after exposure to laser irradiation. When the concentration reached 200 μg/mL, significant 4T1 cell death was observed, with no living cells in the field of view. The findings suggest that the high photothermal conversion efficiency of GIAs could effectively kill tumor cells *in vitro*.

### *In vivo* dual-modal FLI/MRI of GIAs

**FIGURE 3:**
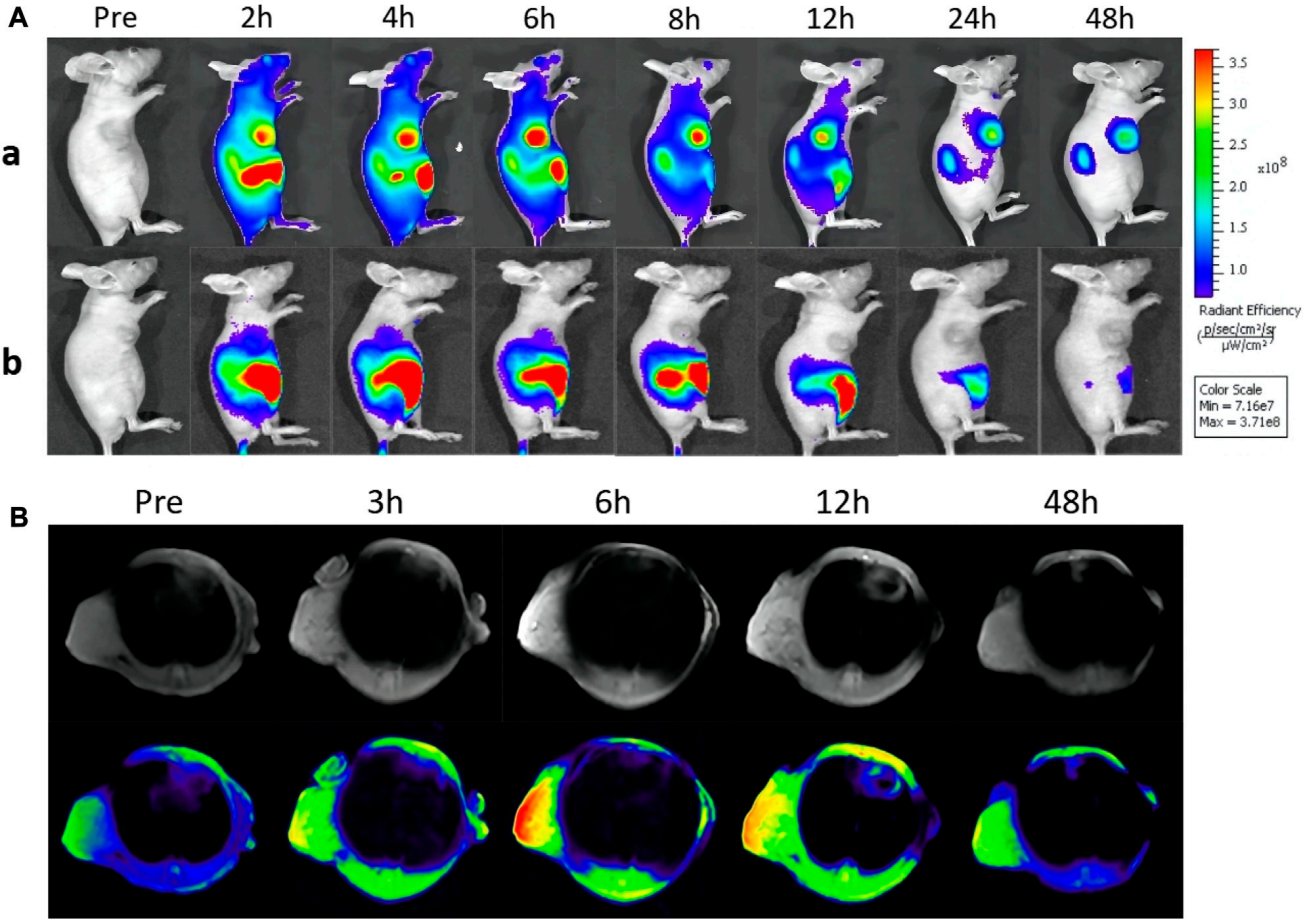
**(A)** *In vivo* fluorescence imaging of mice bearing 4T1 tumors after tail vein injection GIs and ICG. **(B)** MR images of tumor-bearing mice before and after intravenous injection of GIs at different time points (The color from blue to red indicates increasing MR signal intensity).

The *in vivo* fluorescence imaging property of GIAs was investigated in 4T1-tumor-bearing nude mice. After tail vein injection of GIs and free ICG, the fluorescence images were simultaneously recorded by *in vivo* imaging systems at different time points. As shown in Figure 3A, the overall bright fluorescence of mice in the GIAs group confirmed that GIs can quickly assemble with albumin into drug-albumin complexes after entering the bloodstream, thereby remaining in the bloodstream. Subsequently, the fluorescence signal of the tumor region increased substantially and achieved a maximum at 6 h post-injection, maintaining a high level for the following time. This is mainly attributed to the long circulation time of GIAs, EPR effect, and specific uptake of albumin by the tumor. In contrast, almost no signal was detected in the tumor area of mice injected with free ICG due to rapid blood clearance and poor tumor accumulation. Supplementary Figure S3 illustrates *ex vivo* fluorescence images of major organs and tumors harvested from mice at 48 h. As expected, almost no fluorescence was detected in the tumor for the ICG group, whereas bright fluorescence was detected in the GIAs group. Simultaneously, the kidney also showed moderate fluorescence, indicating that the probe was mainly excreted through the kidney. Since GIAs has moderate optical properties at the NIR-II window, the *in vivo* NIR-II imaging performance of GIAs was then examined. As shown in Supplementary Figure S4, after intravenous injection, the NIR-II signal of the tumor region increased substantially and maintained strong fluorescence up to the 48th hour. All these results indicate that GIAs can effectively target and image the tumor.

Considering its remarkable MR contrast performance in solution, the *in vivo* MR imaging ability of GIAs was further evaluated on 4T1-tumor-bearing nude mice. Pre-contrast and contrast MR imaging for the tumor models was performed before and after the injection for various time intervals (3, 6, 12, and 48 h) on a 3.0 T MR system. As shown in Figure 3B, compared with pre-injection, a high T1-weighted MR signal within the tumor can be easily seen over time, with a peak at 6 h post-injection. The enhanced signal lasted for more than 6 hours. This was in good agreement with the *in vivo* fluorescence imaging. Furthermore, the signal-to-noise ratio (SNR) of the tumor region was calculated to quantify the signal change. As shown in Supplementary Figure S5, the SNR rapidly increased to reach the maximum at 6 h post-injection, followed by a signal decrease, which was highly consistent with the MR contrast images. These results indicate that GIAs has excellent MR imaging capacity. Combined with enhanced fluorescence imaging, GIAs exhibits great potential as an FLI/MRI dual-modal probe for cancer imaging.

### *In vivo* PTT of GIAs

**FIGURE 4:**
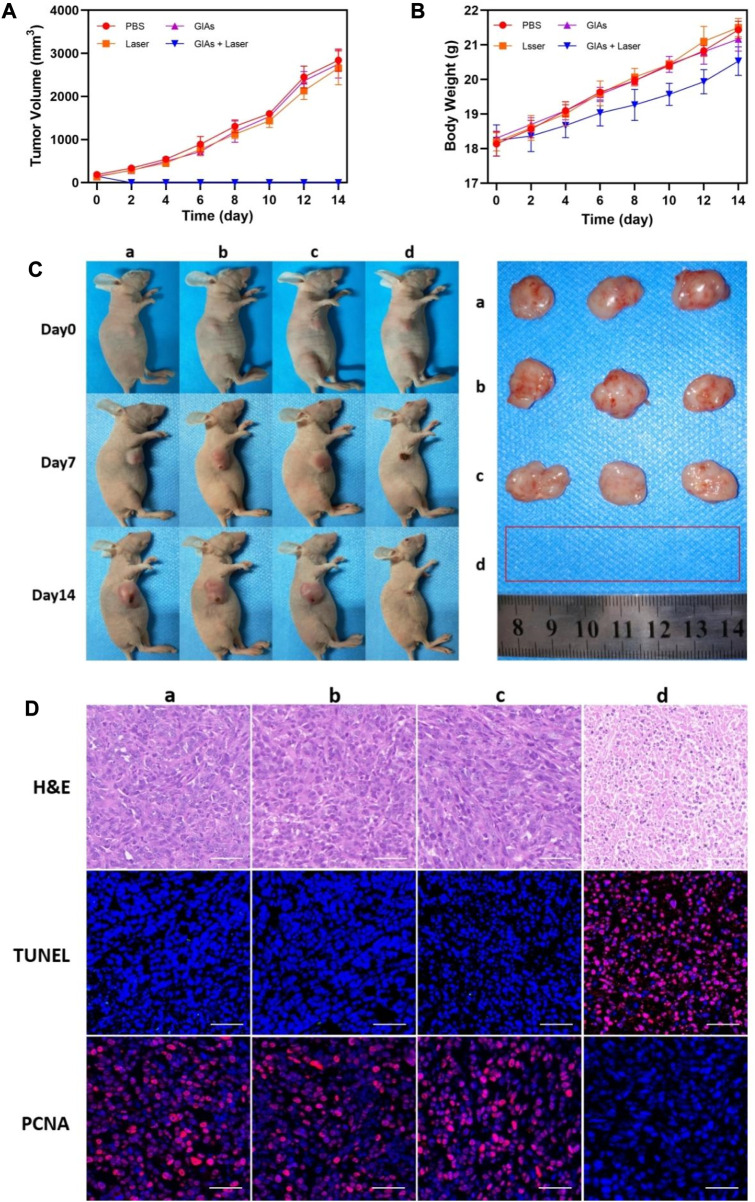
Growth curve of **(A)** tumor volume and **(B)** body weight of mice. **(C)** Photographs of representative mice and extracted tumors. **(D)** H&E, TUNEL and PCNA staining for tumor sections. The red fluorescence indicates the TUNEL or PCNA signal. Scale bar: 50 μm (a, PBS; b, Laser; c, GIAs; d, GIAs + laser).

Encouraged by the above results, a mouse model of 4T1 breast tumor was established to evaluate the therapeutic efficacy of GIAs. The mice were randomly divided into 4 groups: a) PBS only b) Laser only c) GIAs d) GIAs + laser. Tumor volumes and body weights were monitored every other day after laser irradiation. As shown in Figure 4A, the tumors in all three control groups exhibited similar growth speeds, indicating that laser irradiation of tumors and GIAs injection alone do not significantly affect tumor growth. However, the combined treatment of GIAs and laser irradiation resulted in complete tumor ablation without relapse during the experiment, which was attributed to enhanced photothermal conversion efficiency and tumor aggregation. Moreover, no significant differences in body weight were observed in the corresponding groups, suggesting low systemic toxicity in all the treatments (Figure 4B). Figure 4C displays the representative photographs of mice and tumors treated with different methods, which were consistent with the results above.

H&E, TUNEL, and PCNA staining of tumor sections further validated the therapeutic effect on tumors (Figure 4D). H&E staining showed significant coagulation necrosis in the tumor tissues of the GIAs plus laser treatment group, and the nucleus of the tumor cells in the necrotic area shrank, crumbled, and dissolved, which occurred to a lesser extent in other groups. This kind of contrast was more pronounced in TUNEL staining, where high levels of apoptosis were observed in tumor cells in the experimental group, while cells in the control group remained alive. Additionally, PCNA staining was used to demonstrate the proliferation. Similarly, the experimental group showed the least positive signals among all groups, suggesting significant inhibition of tumor proliferation following the combination therapy of GIAs and laser irradiation. These results confirmed the excellent efficacy of the GIAs in cancer photothermal therapy *in vivo*.

### Toxicity evaluation of GIAs *in vivo*

**FIGURE 5:**
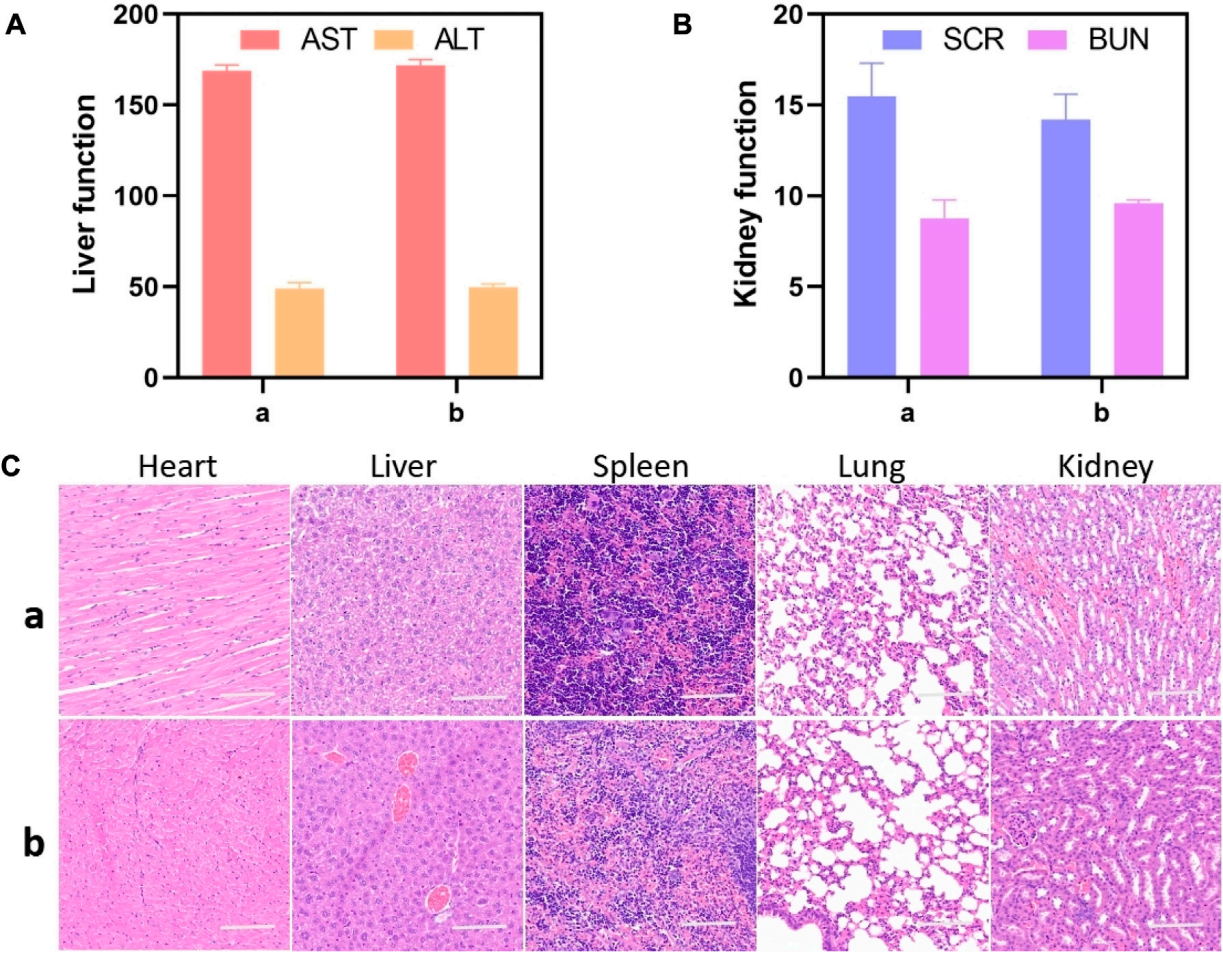
Liver and kidney function markers **(A,B)** and H&E staining images **(C)** of the major organs including the heart, liver, spleen, lung, and kidney of mice after being treated with **(A)** GIAs and **(B)** PBS, respectively. Scale bar: 100 μm.

The *in vivo* toxicology evaluation was conducted on healthy mice by analyzing blood chemistry indexes and histological examination with PBS treated as control group. As illustrated in Figures 5A, B, the important liver and kidney function markers, including aspartate aminotransferase (AST), alanine aminotransferase (ALT), serum creatinine (SCR), and blood urea nitrogen (BUN), were within the normal range. No apparent differences were observed between the GIAs treated group and the control group, suggesting no evident damage of liver and kidney damage after GIAs treatment. H&E staining images revealed no evident tissue damage, inflammation, or lesions of each organ in both the treatment group and the control group, as shown in Figure 5C. All the above results suggested GIAs possessed excellent biocompatibility for *in vivo* application.

## Conclusion

This paper describes the design and synthesis of a novel multifunctional probe GI for FL/MR dual-modal imaging-guided photothermal therapy of cancer. GIs can efficiently assemble with endogenous albumin to form GIAs and self-deliver to the tumor region. In *in vitro* experiments, the prepared GIAs displayed synergistic enhancement of fluorescence emission, MR contrast, and photothermal efficiency. *In vivo* experiments revealed prominent NIR-I/NIR-II/MR imaging and photothermal therapy performance on tumor-bearing mice. Additionally, no potential toxicity was observed in cytotoxicity, serum biochemistry, and histological analyses. Overall, this work provides a simple and feasible strategy for the preparation of a synergistically enhanced multifunctional probe, which holds great potential for cancer theranostics.

## Data Availability

The original contributions presented in the study are included in the article/Supplementary Material, further inquiries can be directed to the corresponding authors.
