# Supplementary material for: Novel albumin-binding multifunctional probe for synergistic enhancement of FL/MR dual-modal imaging and photothermal therapy
Source: Front Chem. 2023 Aug 1;11:1253379. doi: 10.3389/fchem.2023.1253379 (PMC10427858; doi:10.3389/fchem.2023.1253379)
Supplement: Supplementary file 1 [file Table1.DOCX]

Supplementary Material

**Novel Albumin-Binding Multifunctional Probe for Synergistic Enhancement of FL/MR Dual-Modal Imaging and Photothermal Therapy**

Cheng Yu^1^, Zhuyuan Ding^1^, Huan Liu^1^, Yulu Ren^1^, Minping Zhang^1^, Qiuling Liao^1^, Tao Luo^1^, Lujing Gao^1^, Shiyi Lyu^1^, Huiwen Tan^1^, Linan Hu^2^, Zhu Chen^1^*, Pengfei Xu^34^* and Enhua Xiao^1^*

^a^ Departments of Radiology, The Second Xiangya Hospital, Central South University, Changsha, Hunan 410011, P.R. China;

^b^ Department of Radiology, Zhuzhou Central Hospital, Zhuzhou, Hunan 412000, P.R. China;

^c^ Translational Pharmaceutical Laboratory, Jining First People’s Hospital, Shandong First Medical University, Jining 272000, China;

^d^ Institute of Translational Pharmacy, Jining Medical Research Academy, Jining 272000, China.

**Experimental**

**Equipment and Methods**

UV-vis absorption spectra were measured on a Hitachi UV-2600 spectrophotometer (Japan). Photoluminescence spectra were recorded on an Edinburgh FLS1000 spectrofluorometer (UK). The absorbance of the CCK8 assay was recorded on a Thermo Fisher microplate reader (USA) at a wavelength of 490 nm. In vivo MR imaging and T1 relaxation time measurements were performed on a 3.0 T uMR 790 MRI system (United, China). Fluorescence imaging was performed on a small animal in vivo imaging system (IVIS Spectrum BL, PerkinElmer, USA). Electrospray ionization mass spectra (ESI-MS) were collected on an LTQ Orbitrap Velos Pro high-resolution LC/MS instrument (Thermo Fisher, USA).

**Preparation of GI**

The synthesis route for GI is shown in Fig. S1. EB-Lys was prepared according to the literature method with a slight modification. EB-DOTA was produced by a simple conjugation between DOTA-NHS and EB-Lys, which was further hydrolyzed with TFA to remove the Boc protecting group. Then, Compound 3 was prepared from the conjugation of ICG-NHS with Compound 2. The corresponding Gd(III) complex was formed by reacting Compound 3 with an equimolar amount of gadolinium chloride in water.

**Synthesis of Compound 2**

DOTA-NHS (50 mg, 1 eq) was placed in 10 mL anhydrous N, N-dimethylformamide (DMF) and was stirred with EB-Lys (77 mg，1.1 eq) with the addition of DIPEA (3 eq). The mixture was stirred at room temperature for 4 hours. Subsequently, DMF was removed under high vacuum and the residue was redissolved in 10% TFA in DCM (v/v) at room temperature. The reaction was monitored by HPLC analysis and was completed in 2 hours. The mobile phases (A) demineralized water and (B) acetonitrile were acidified to pH 3 with trifluoroacetic acid. Gradient elution was performed as follows: 10% of B, 0-3 min;10-90% of B, 3-12 min; 90% of B, 12-14 min; 90-10% of B, 14-16min; 10% of B, 16-18min. The product was then purified by Pro-HPLC. MS analysis confirmed a mass of 1055.70 [M-H]- with an isolated yield of 61% (64 mg).

**Synthesis of Compound 3**

ICG-NHS (16 mg, 1 eq) was placed in 3mL anhydrous N, N-dimethylformamide (DMF) and was stirred with Compound 2 (21 mg，1 eq) with the addition of DIPEA (3 eq). Then, the mixture was stirred under an N2 atmosphere at room temperature. The reaction was monitored by HPLC analysis and was completed in 4 hours. The product was then purified by Pro-HPLC. MS analysis confirmed a mass of 1767.70 [M-2H]-with an isolated yield of 47% (17 mg).

**Synthesis of Compound 4**

Compound 3 (17mg, 1 eq) was dissolved in water (5 mL) and gadolinium(III) chloride hexahydrate (3.8 mg, 1 eq) was added. The mixture was stirred at room temperature for 4 h, during which the pH of the solution was periodically adjusted to 7.0-7.5 with NaOH (1.0 M). Water was removed by evaporation, and the remaining oily product was purified by Pro-HPLC. MS analysis confirmed a mass of 1946.70 [M+Na]+with an isolated yield of 63% (12 mg).

**Cell Culture and Uptake**

Mouse breast cancer 4T1 cells were cultured in RPMI-1640 medium containing 10% fetal bovine serum and 1% penicillin/streptomycin at 37 ℃ under 5% CO2. The cells were seeded at a density of 1 × 104 per well into a 6-well plate. After 24 h of growth, cells were incubated with GIAs solution at a concentration of 25 μg/ml. Then, the cell nuclei were marked with DAPI. Finally, fluorescent images were acquired by an inverted fluorescence microscope (Leica DMIL LED+EC3) and processed using imaging software. Fluorescence signals of GIAs and DAPI probe were collected in the various fluorescent channels that were recommended by the manufacturer.

**Cytotoxicity Assay**

The cytotoxicity of GIAs was evaluated by a CCK-8 cell proliferation kit (Bimake, USA). 4T1 cells were seeded into a 96-well plate at a density of 5000 cells per well, and 5 replicate wells were set up. After overnight incubation, cells were cultured with GIAs at different concentrations (12.5, 25, 50, 100, and 200 μg/mL). After 24 h, cells were washed with PBS and 100 µL fresh medium containing 10% CCK-8 was added to each well. Finally, the 96-well plates were placed in a microplate reader (Thermo Fisher, USA) to measure the absorbance at 450nm. The experiment was repeated in triplicate.

***In Vitro* PTT**

The in vitro photothermal effect was evaluated by live/dead cell staining analysis. 4T1 cells were seeded in a 24-well plate at a density of 1 x 104 cells per well and incubated with different concentrations of GIAs suspensions (0, 50, 100, and 200 μg/mL). After irradiation with a NIR laser (808nm, 0.5W/cm2) for 5 min, fluorescein diacetate (FDA) and propidium iodide (PI) were used to stain live and dead 4T1 cells for visualization. Fluorescence images of the stained cells were obtained using an inverted fluorescence microscope.

**Tumor Model**

Female Balb/c nude mice (6 weeks old, Department of Experimental Animals of Central South University) were used to establish 4T1 xenograft tumor models. 4T1 cells suspended in PBS were subcutaneously injected into the right armpit of each mouse. The mice were used for *in vivo* experiments when the tumor volume reached approximately 100 mm3. All animal procedures were performed in accordance with the Guidelines for Care and Use of Laboratory Animals of Central South University and approved by the Animal Ethics Committee, The Second Xiangya Hospital, Central South University, China.

***In Vivo* FL Imaging and Biodistribution**

The 4T1 tumor-bearing mice were randomly divided into two groups (n=3). Subsequently, equimolar solutions of GIs and free ICG were intravenously injected into the tail of the mice. Fluorescent pictures at different time points were captured using a NIR-I and NIR-II *in vivo* imaging system under inhalation anesthesia. At 48 h post-administration, the mice were immediately euthanized. The tumors and major organs of mice were excised to evaluate their fluorescence intensities.

**MRI analysis**

The T_1_ relaxation times of GIAs and GIs were measured by a 3.0 T MRI scanner with a Gd(III) concentration of 0.05, 0.1, 0.2, 0.4, and 0.8 mM, respectively. Then, longitudinal relaxivity (r1) was calculated as the slope of the plot of 1/T1 versus the concentration of Gd(III).

Subsequently, contrast enhancement of GIAs was preliminarily evaluated in 4T1 tumor-bearing mice. Three mice were anesthetized by intravenous injection of 2% sodium pentobarbital solution (0.1 mL/20 g). Then, 0.1 mmol Gd/kg of contrast agent was intravenously administered to anesthetized mice via the tail vein. MR images were acquired using a fast spin-echo sequence (FSE) with the following parameters: TR/TE = 420 ms/11.98 ms, slice thickness = 1 mm, FOV = 40×80. The images were analyzed on a United imaging workstation, and the signal intensity (SI) of the tumor was measured.

***In Vivo*** **PTT**

The 4T1 tumor-bearing nude mice were used for *in vivo* PTT. The mice were divided randomly into 4 groups (n=4 in each group): (a) PBS only (b) Laser only (c) GIAs (1mg/ml) (d) GIAs (1mg/ml) + laser. Each group was intravenously injected with 200 µL of solution. FL/MR imaging was first performed prior to PTT for outlining the tumor and further determining the range of the irradiation field. After 12 h post-injection, the tumor sites of mice in groups 2 and 4 were irradiated with 808 nm laser (1.0W/cm^2^, 10 min). The next day, one mouse from each group was randomly selected and sacrificed, and the tumor tissues were collected. The tumor tissues were stained with H&E, TdT-mediated dUTP Nick-End Labeling (TUNEL), and Proliferating Cell Nuclear Antigen (PCNA). Animal weight and tumor volume were recorded every 2 days.

***In vivo* systemic toxicity evaluation**

The healthy mice were randomly divided into two groups (n=3) to assess the systemic toxicity of GIAs. Subsequently, equal concentrations of GIs and PBS solutions (0.1 mmol/kg) were intravenously injected into the tails of mice. 24 hours after injection, three mice were euthanized, and their major organs (heart, liver, spleen, lung, and kidney) and eyeball blood were collected. Eyeball blood was used to evaluate the liver and kidney function, including indicators such as aspartate aminotransferase (AST), alanine aminotransferase (ALT), serum creatinine (SCR), and blood urea nitrogen (BUN). The main organ tissues were sectioned and stained with Hematoxylin-Eosin (H&E). The histological sections were analyzed to assess *in vivo* toxicity.

**Statistical analysis**

All statistical analyses and graph generation were performed using GraphPad Prism 9 software. The statistical significance was determined at the level of p < 0.05.


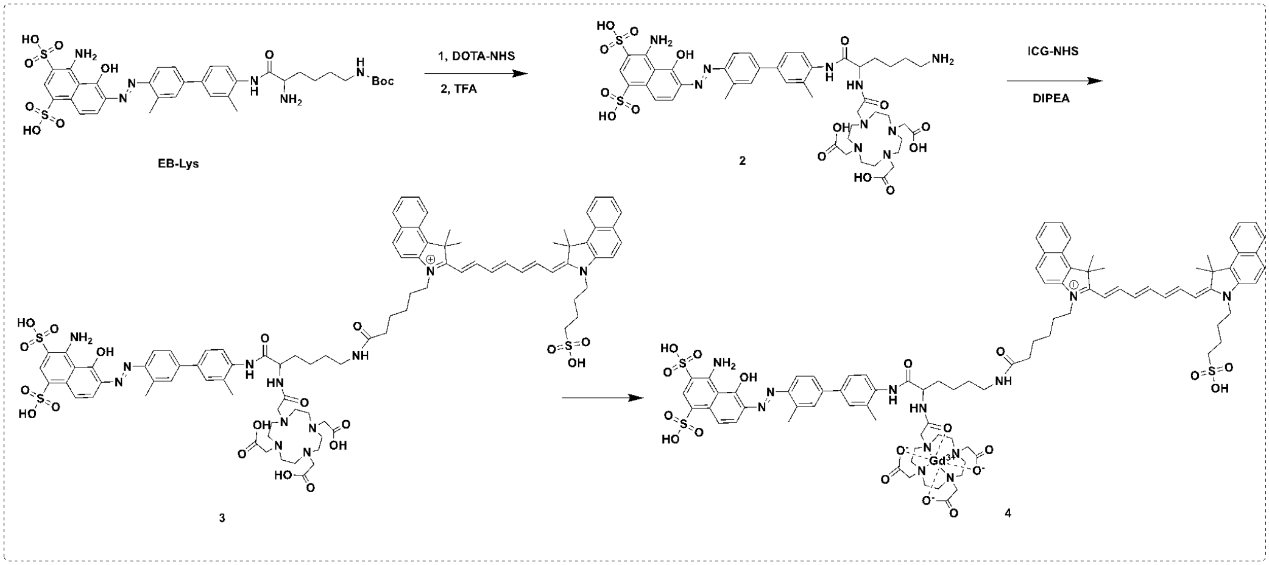


**Figure S1.** Synthesis route of GI.


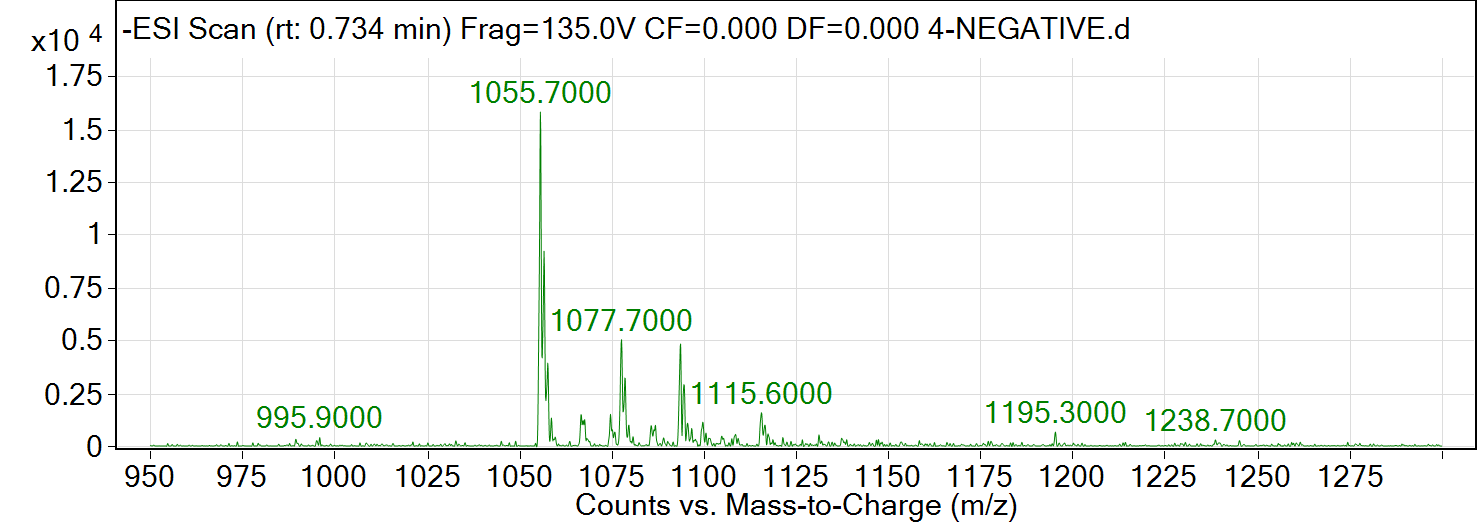


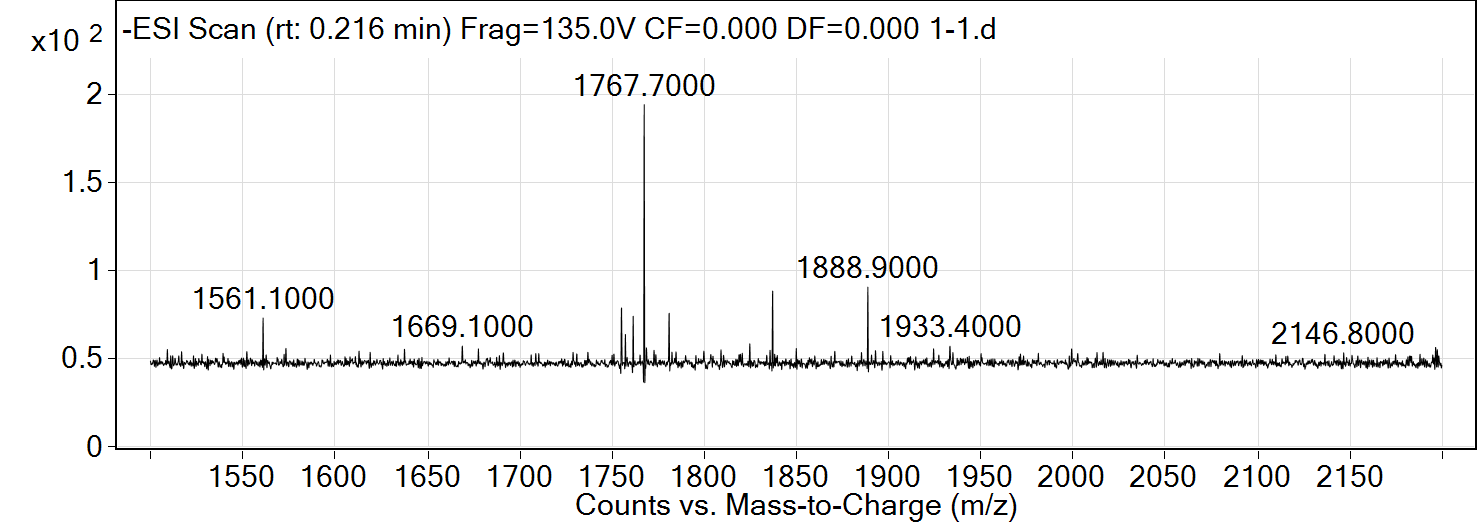


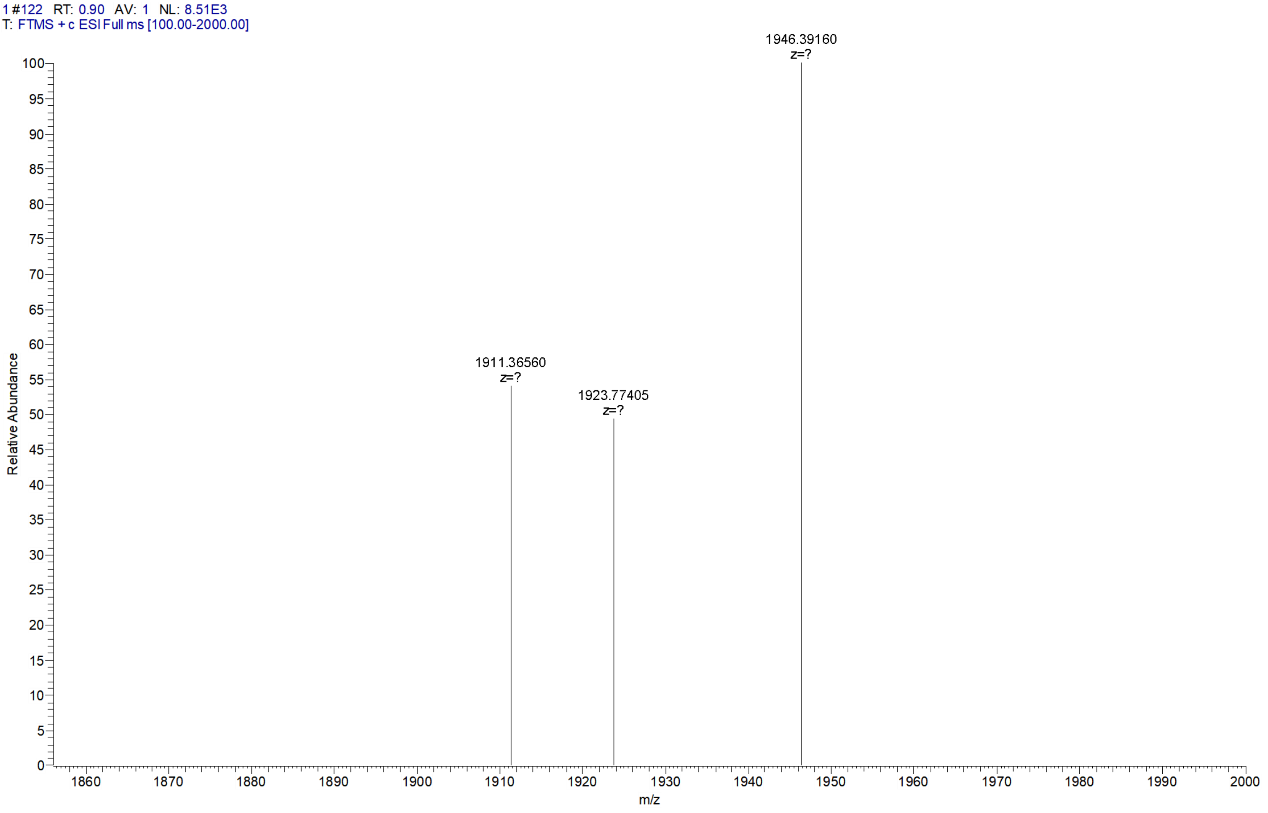


**Figure S2.** MS spectroscopy of GI.


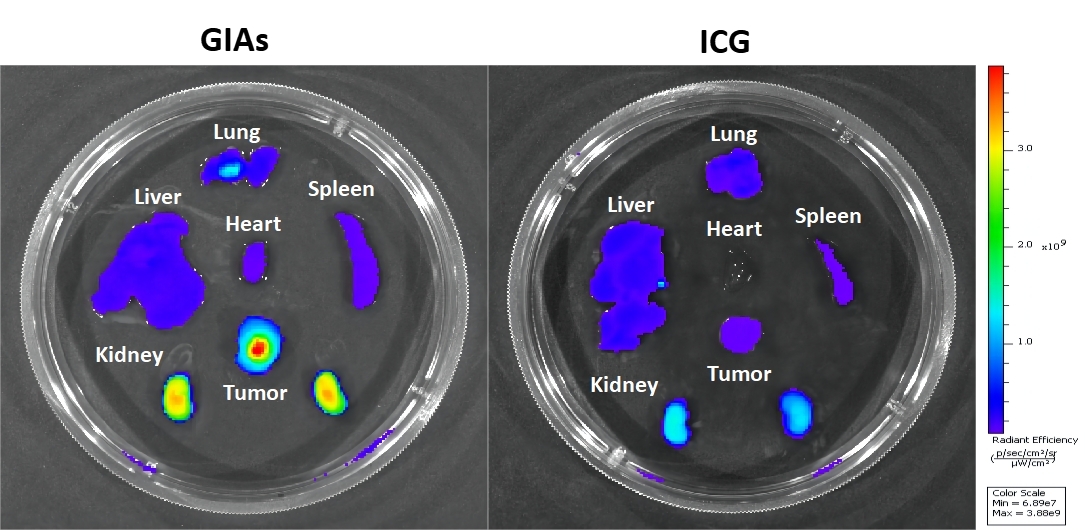


**Figure S3.** Fluorescence images of major organs and tumors after 48 h post-injection of GIs and free ICG.


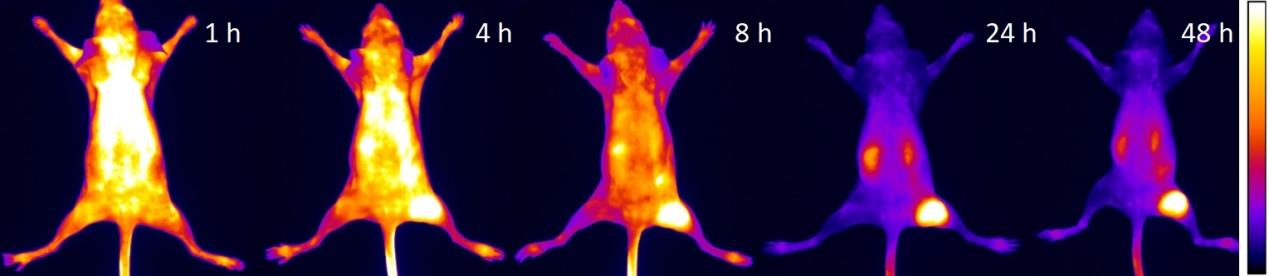


**Figure S4.** NIR-II imaging of mice bearing 4T1 tumors after tail vein injection of GIs.


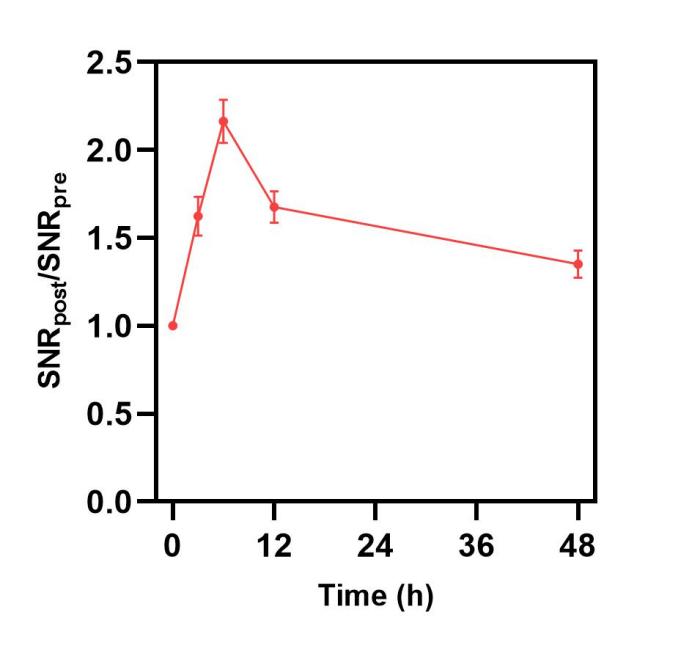


Figure S5. Quantitative analysis of signal changes (SNR ratio) in tumor versus treating time.
